# Anti-Interleukin-16-Neutralizing Antibody Attenuates Cardiac Inflammation and Protects against Cardiac Injury in Doxorubicin-Treated Mice

**DOI:** 10.1155/2021/6611085

**Published:** 2021-04-17

**Authors:** Jianwei Zhang, Zicong Yang, Zhishan Liang, Mengjie Wang, Changxing Hu, Chao Chang, Lei Shi, Qingwei Ji, Ling Liu

**Affiliations:** ^1^Department of Cardiology, Beijing Anzhen Hospital, Capital Medical University, Beijing Institute of Heart, Lung, and Blood Vessel Diseases, The Key Laboratory of Remodeling-related Cardiovascular Disease, Ministry of Education, Beijing 100029, China; ^2^Department of Cardiology, The People's Hospital of Guangxi Zhuang Autonomous Region, Nanning, China; ^3^Department of Cardiology, Handan First Hospital, Handan, Hebei, China

## Abstract

**Background:**

Interleukin-16 (IL-16) is an important inflammatory regulator and has been shown to have a powerful effect on the regulation of the inflammatory response. Cardiac inflammation has been reported to be closely related to doxorubicin- (DOX-) induced cardiac injury. In this study, the role of IL-16 in DOX-induced cardiac injury and the possible mechanisms were examined.

**Methods:**

Cardiac IL-16 levels were first measured in DOX- or saline-treated mice. Additionally, mice were pretreated with the anti-IL-16-neutralizing antibody (nAb) or isotype IgG for 1 day and further administered DOX or saline for 5 days. Then, cardiac injury, cardiac M1 macrophage levels, and cardiomyocyte apoptosis were analyzed. The effects of the anti-IL-16 nAb on macrophage differentiation and cardiomyocyte apoptosis were also investigated in vitro.

**Results:**

DOX administration increased IL-16 expression in cardiac macrophages compared with that of saline treatment. The anti-IL-16 nAb significantly decreased serum levels of lactate dehydrogenase (LDH), myocardial-bound creatine kinase (CK-MB), and cardiac troponin T (cTnT) and elevated cardiac function in DOX-induced mice. Treatment with the anti-IL-16 nAb also reduced p65 pathway activation, decreased M1 macrophage-related marker and cytokine expression, and protected against cardiomyocyte apoptosis in DOX-induced mice. In cell studies, the anti-IL-16 nAb also reduced DOX-induced M1 macrophage differentiation and alleviated apoptosis in cardiomyocytes cocultured with macrophages.

**Conclusions:**

The anti-IL-16 nAb protects against DOX-induced cardiac injury by reducing cardiac inflammation, and IL-16 may be a promising target to prevent DOX-related cardiac injury.

## 1. Introduction

As a drug that has been widely used in clinical chemotherapy, doxorubicin (DOX) has slowly been withdrawn as a frontline treatment due to severe cardiotoxicity and cardiac injury and further grave clinical consequences [1, 2]. A variety of pathological injury factors, including inflammatory response, oxidative stress, excessive apoptosis, and energy metabolic failure, have been found to be involved in the progression of DOX-induced cardiotoxicity and cardiac injury [1–4]. Studies have shown that immune cell activation and the release of numerous inflammation-related substances play crucial roles in DOX-induced cardiotoxicity and cardiac injury [2–4].

Interleukin-16 (IL-16) is an important proinflammatory cytokine that can be secreted by immune cells, including activated T lymphocytes and macrophages, as well as nonimmune cells, such as mast cells and epithelial cells [5–7]. IL-16 was originally considered to be a chemokine associated with CD4+ T lymphocytes; however, there have now been a considerable number of studies indicating that IL-16 also mediates the chemotactic activity and/or differentiation of macrophages, monocytes, and mast cells [6–12]. IL-16 is involved in signal regulation by activating a variety of signaling pathways, including the phosphatidylinositol 3-kinase (PI3K), nuclear factor kappa-B (NF-*κ*B) p65, stress-activated protein kinase (SAPK), and mitogen-activated protein kinase (MAPK) pathways [13–15].

IL-16 has been demonstrated to be involved in the progression of a variety of diseases, including colitis, allergic reactions, pneumonia, tumors, and spinal cord injuries, by regulating the immune and inflammatory responses [5, 7, 8, 10–13]. However, the association between IL-16 and cardiovascular disease has not been thoroughly reported. In an early study, the IL-16 TG/GG genotypes of rs11556218 T/G were reported to significantly increase the incidence of coronary artery disease [16]. Another study reported that elevated IL-16 was associated with a reduced incidence of cardiovascular events in patients with carotid atherosclerosis [17]. Circulating IL-16 levels were found to be elevated in chronic heart failure (CHF) patients with preserved left ventricular (LV) ejection fraction (LVEF), while IL-16 overexpression increased cardiac macrophage infiltration and exacerbated cardiac fibrosis in angiotensin II- (Ang II-) infused mice, and IL-16 neutralization had the opposite effects [18]. However, whether IL-16 is involved in DOX-induced cardiac injury via regulating cardiac inflammation remains unclear. In this study, we examined the effect of an anti-IL-16-neutralizing antibody (nAb) on DOX-induced cardiac injury and inflammation and attempted to explain its possible mechanisms.

## 2. Materials and Methods

### 2.1. Mice and Treatments

Male wild-type (WT) mice with a C57BL/6 background were ordered from Beijing Vital River Laboratory Animal Technology, housed in a pathogen-free mouse room (temperature: 23 ± 1°C; 12 hours light/12 hours dark) at Beijing Anzhen Hospital and received water ad libitum from the Animal Care Facility Service. Mice aged 9-10 weeks and weighing 24.5-25.5 g were used in this study. First, the mice were administered 15 mg/kg DOX by a single intraperitoneal injection, and mice were administered saline as a control (*N* = 10 for each group). Then, cardiac IL-16 expression was measured 5 days later. Additionally, the mice were pretreated with 200 *μ*g of mouse anti-IL-16 nAb or the same amount of isotype IgG for 1 day and then treated with DOX or saline for 5 days (*N* = 10 for each group) [18]. The body weight (BW) was measured at different time points before and after DOX administration, and heart weight (HW) and tibia length (TL) were measured after the mice were euthanized. The use of mice and the study were approved by the Ethics Committee of Beijing Anzhen Hospital.

### 2.2. Analysis of Cardiac Function

After the mice were anesthetized using 1.5%-2% isoflurane, echocardiography was performed, and M-mode images of the left ventricle at the papillary muscle level were recorded for 5 cardiac cycles. Then, the heart rate (HR), LVEF, and fractional shortening (FS) data were analyzed. Next, a microtip catheter transducer was inserted into the carotid artery and further inserted into the left ventricle, and readings of the maximal slopes of the systolic pressure increment (+dp/dt max) and the diastolic pressure decrement (-dp/dt max) in 10 cardiac cycles were collected and analyzed.

### 2.3. Cell Experiments and Treatments

CTLL-2 T lymphocytes, RAW264.7 macrophages, DC2.4 dendritic cells, and HL-1 cardiomyocytes were all purchased from ATCC (USA) and cultured in RPMI 1640 medium containing 10% fetal bovine serum and antibiotics at 37°C and 5% CO_2_. First, the cells were treated with 1 *μ*M DOX or saline for 12 hours, and then, the IL-16 mRNA levels in each group of cells were measured. Anti-IL-16 nAb- or IgG-treated RAW264.7 macrophages were administered DOX or saline, and nuclear p-p65 expression, CD80 and inducible nitric oxide synthase (iNOS) mRNA expression levels, and CD80 protein expression in macrophages were measured. Furthermore, HL-1 cardiomyocytes were cocultured with RAW264.7 macrophages, and the protein levels of Bax, Bcl-2, and cleaved caspase-3 in HL-1 cardiomyocytes were measured.

### 2.4. Analysis of Serum IL-16 and Cardiac Injury Marker Levels

Serum was obtained from each blood sample, and a mouse enzyme-linked immunosorbent assay (ELISA, NeoBioscience, China) kit was used to determine serum IL-16 levels according to the manufacturer's instructions. Additionally, serum cardiac injury markers, including lactate dehydrogenase (LDH), myocardial-bound creatine kinase (CK-MB), and cardiac troponin T (cTnT), were analyzed using kits according to the experimental procedure provided by the manufacturer.

### 2.5. Nuclear and Cytoplasmic Separation and Western Blotting

The cytoplasm and nuclei in LV tissue and RAW264.7 macrophages were isolated using a nuclear separation kit (Njjcbio) according to a previously described protocol [19]. Briefly, homogenate was collected from LV tissue and cells that were lysed in lysis buffer and further centrifuged at 1000 × g for 10 minutes. The pellets in the bottoms of the tubes contained the nuclei, and the supernatant contained the cytoplasm.

After the cytoplasm, nuclei, LV tissue, and HL-1 cardiomyocytes were, respectively, lysed in RIPA lysis buffer containing 10% protease inhibitors and 10% phosphatase inhibitors, the total protein was obtained and quantitated using a BCA Protein Assay Kit. After separation by electrophoresis on 10% SDS polyacrylamide gels, the proteins were transferred to PVDF membranes. Then, the protein expression of IL-16 (Abcam), Bax, Bcl-2, cleaved caspase-3, caspase-3, and GAPDH (all three from GeneTex) in total LV tissue, the protein expression of p-p65 and GAPDH (both from Abcam) in the cytoplasm, the protein expression of p-p65 and PCNA (GeneTex) in the nuclei, and the protein expression of Bax, Bcl-2, cleaved caspase-3, caspase-3, and GAPDH in HL-1 cardiomyocytes were measured using primary antibodies as indicated in parentheses. After further incubation with the secondary antibodies, the target proteins were detected and analyzed.

### 2.6. Real-Time Quantitative Polymerase Chain Reaction (RT-qPCR)

Total RNA was isolated from lysed LV tissue and cells and was further converted to cDNA using a cDNA synthesis kit (Thermo Fisher). Then, PCR amplification was performed using the SYBR Green PCR master mix (Vazyme) to determine target mRNA expression. In this study, expression of the target mRNAs IL-16, tumor necrosis factor *α* (TNF-*α*), IL-1*β*, IL-6, IL-17, IL-18, interferon-*γ* (IFN-*γ*), monocyte chemotactic protein-1 (MCP-1), CD80, and iNOS was measured and normalized to the GAPDH mRNA levels. All gene primers used in this study are listed in Table 1.

### 2.7. Histological Analysis

Fresh hearts and RAW264.7 macrophages were incubated in 4% paraformaldehyde for 4 days, embedded in paraffin, and sectioned at thicknesses of 4-6 *μ*m. Then, the vacuolization of cardiomyocytes was measured by histopathological analysis using hematoxylin and eosin (HE) staining. Apoptotic cardiomyocytes were measured using terminal deoxynucleotidyl transferase-mediated dUTP nick end-labeling (TUNEL) staining. Anti-CD80 and anti-iNOS antibodies were used for immunohistochemical staining to detect cardiac CD80 and iNOS protein expression. Additionally, immunofluorescence staining was performed using an anti-IL-16 antibody to detect cardiac IL-16 expression. Double immunofluorescence staining using anti-IL-16 and anti-F4/80 antibodies and anti-CD80 and F4/80 antibodies was performed to measure IL-16 expression in total and M1 macrophages.

### 2.8. Statistical Analysis

All data in this study are presented as the mean ± SD and were analyzed using GraphPad Prism 8. For the data with homogeneous of variance and normal distribution, differences between 2 groups were compared using Student's *t*-test and differences among 3 or more groups were analyzed using ANOVA, followed by Tukey's multiple comparisons test. For the data with heterogeneity of variance or abnormal distribution, the differences among different groups were analyzed using a nonparametric test. A *p* value of less than 0.05 was considered indicative of a significant difference between the mean values of the groups.

## 3. Results

### 3.1. DOX Administration Promotes IL-16 Release from Cardiac Macrophages in Mice

**Figure 1:**
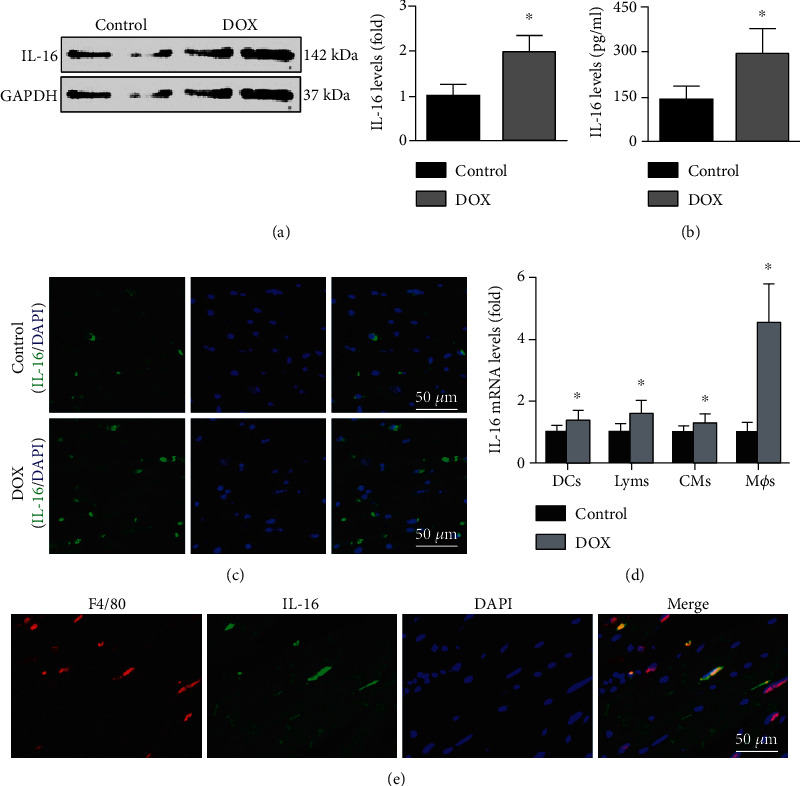
Effects of DOX on cardiac IL-16 expression. (a, b) Cardiac IL-16 expression and serum IL-16 levels were measured in the control and DOX groups (nonparametric test). (c) Cardiac IL-16 expression in the 2 groups was determined by immunofluorescence staining (200x). (d) Effects of DOX on IL-16 mRNA expression in CTLL-2 T lymphocytes (Lyms), RAW264.7 macrophages (Møs), DC2.4 dendritic cells (DCs), and HL-1 cardiomyocytes (CMs) (Student's *t*-test). (e) Double immunofluorescence staining with anti-F4/80 and anti-IL-16 in DOX-induced mice (200x). *N* = 5 in each group. ^∗^*p* < 0.05 vs. the control group.

The western blot results showed that DOX increased both cardiac IL-16 expression and serum IL-16 levels compared with the control condition (Figure 1(a)). Immunofluorescence staining also showed increased cardiac IL-16 expression (Figure 1(b)). DOX also increased IL-16 mRNA levels in dendritic cells, T lymphocytes, cardiomyocytes, and especially in macrophages (Figure 1(c)). Double immunofluorescence staining showed that IL-16 was produced by cardiac macrophages (Figure 1(d)).

### 3.2. Anti-IL-16 nAb Alleviates DOX-Induced Cardiac Injury and Dysfunction in Mice

**Figure 2:**
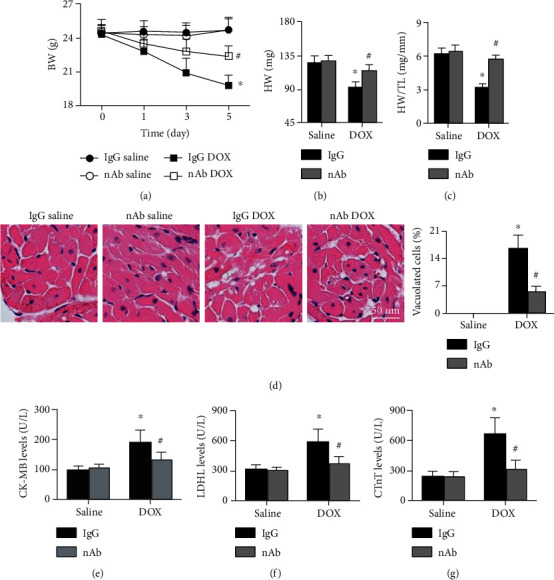
Effects of anti-IL-16 nAb on DOX-induced cardiac injury. (a) BW changes at different time points in the four groups (ANOVA). (b, c) HW and HW/TL ratios in each group (ANOVA). (d) HE staining and cardiomyocyte vacuolization analysis of each group (200x, nonparametric test). (e-g) Serum LDH, CK-MB, and cTnT levels were detected (ANOVA). *N* = 5-10 for each group. ∗*p* < 0.05 vs. the IgG saline group. ^**#**^*p* < 0.05 vs. the IgG DOX group.

The BWs were significantly decreased in DOX-induced mice, but these changes were reversed by the anti-IL-16 nAb (Figure 2(a)). The anti-IL-16 nAb also increased the HWs and the HW/TL ratios in DOX-induced mice (Figure 2(b)). Lower cardiomyocyte vacuolization percentages were observed in the IgG DOX group than in the nAb DOX group (Figure 1(c)). The same trends in serum cardiac injury markers, including LDH, CK-MB, and cTnT, were observed (Figure 2(d)). In addition, DOX administration decreased HR, LVEF, and FS, and the anti-IL-16 nAb increased HR, LVEF, and FS in DOX-induced mice, and similar trends in +dp/dt and -dp/dt were observed. The results are listed in Table 2.

### 3.3. Anti-IL-16 nAb Inhibits M1 Macrophage Differentiation in DOX-Induced Mice

**Figure 3:**
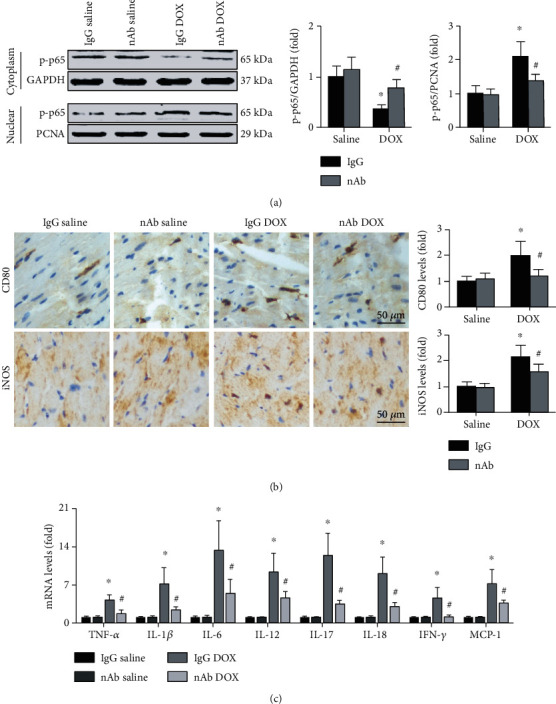
Effects of anti-IL-16 nAb on DOX-induced M1 macrophage differentiation. (a) Protein expression of p-p65 in both the cytoplasm and nucleus was detected in the four groups (nonparametric test). (b) Cardiac expression of CD80 (brown granules) and iNOS (brown granules) was detected by immunohistochemical staining (200x, ANOVA). (c) Cardiac mRNA levels of TNF-*α*, IL-1*β*, IL-6, IL-17, IL-18, IFN-*γ*, and MCP-1 were detected in each group by RT-qPCR (ANOVA). *N* = 5 for each group. ^∗^*p* < 0.05 vs. the IgG saline group. ^**#**^*p* < 0.05 vs. the IgG DOX group.

First, p65 phosphorylation was measured, and the results showed that DOX administration decreased cytoplasmic p-p65 expression and increased nuclear p-p65 expression, while the anti-IL-16 nAb increased cytoplasmic p-p65 levels and decreased nuclear p-p65 levels in DOX-induced mice (Figure 3(a)). Immunohistochemical staining showed that the anti-IL-16 nAb decreased cardiac CD80 and iNOS expressions in DOX-induced mice (Figure 2(b)). Similar trends in the cardiac mRNA expression of M1 macrophage-related cytokines, including TNF-*α*, IL-1*β*, IL-6, IL-17, IL-18, IFN-*γ*, and MCP-1, were observed (Figure 3(c)).

### 3.4. Anti-IL-16 nAb Protects against Cardiomyocyte Apoptosis in DOX-Induced Mice

**Figure 4:**
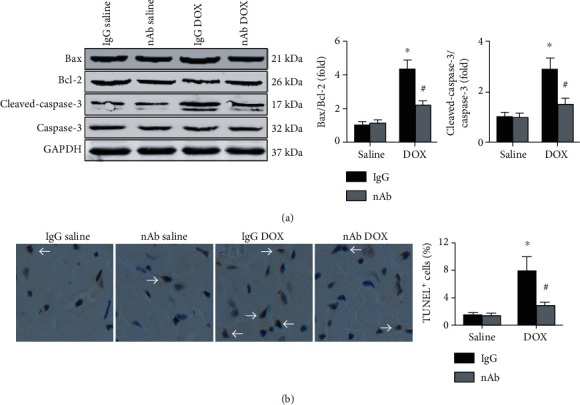
Effects of anti-IL-16 nAb on DOX-induced cardiomyocyte apoptosis. (a) Protein expression of Bax, Bcl-2, cleaved caspase-3, and caspase-3 were measured in the left ventricle, and the Bax/Bcl-2 and cleaved caspase-3/caspase-3 ratios were analyzed (ANOVA). (b) Cardiac TUNEL-positive cells were measured and analyzed. *N* = 5 for each group (200x, nonparametric test). ^∗^*p* < 0.05 vs. the IgG saline group. ^**#**^*p* < 0.05 vs. the IgG DOX group.

Cardiac apoptosis-associated proteins were measured, and the results showed that the anti-IL-16 nAb decreased both the Bax/Bcl-2 ratios and cleaved caspase-3/caspase-3 ratios in DOX-induced mice (Figure 4(a)). Furthermore, cardiomyocyte apoptosis was measured, and the results showed that DOX elevated the number of TUNEL-positive cells, which was decreased by the anti-IL-16 nAb (Figure 4(b)).

### 3.5. Anti-IL-16 nAb Inhibits DOX-Induced M1 Macrophage and Cardiomyocyte Apoptosis In Vitro

**Figure 5:**
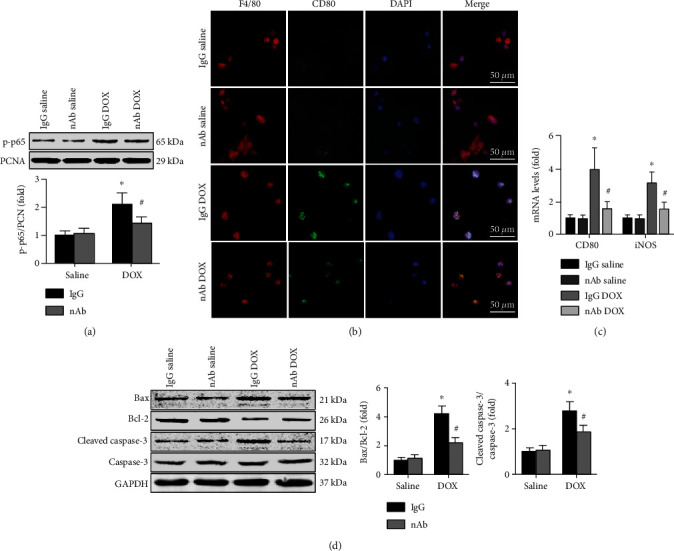
Effects of anti-IL-16 nAb on DOX-induced M1 macrophage differentiation and cardiomyocyte apoptosis in vitro. (a) Nuclear p-p65 expression in RAW264.7 macrophages was detected (ANOVA). (b) Double immunofluorescence staining of anti-F4/80 and anti-IL-16 in DOX-treated mice (200x, nonparametric test). (c) The mRNA expression of CD80 and iNOS was detected (ANOVA). (d) Protein expression of Bax, Bcl-2, cleaved caspase-3, and caspase-3 was measured and the ratios of Bax/Bcl-2 and cleaved caspase-3/caspase-3 (ANOVA). *N* = 5 for each group. ^∗^*p* < 0.05 vs. the IgG saline group. ^**#**^*p* < 0.05 vs. the IgG DOX group.

Nuclear p65 activation in RAW264.7 macrophages was measured, and the results showed that the anti-IL-16 nAb reversed the DOX-induced increase in p-p65 expression (Figure 5(a)). More CD80 expression was observed in the IgG DOX group than in the anti-IL-16 nAb DOX group (Figure 5(b)). Similar trends in the mRNA expression of CD80 and iNOS in RAW264.7 macrophages were observed (Figure 5(c)). In HL-1 cardiomyocytes, DOX administration increased both the Bax/Bcl-2 ratios and cleaved caspase-3/caspase-3 ratios, and the anti-IL-16 nAb exerted the opposite effect in the absence of DOX (Figure 5(d)).

## 4. Discussion

The role of IL-16 in cardiovascular disease has rarely been reported, and the aim of this study was to examine the effects of IL-16 on DOX-induced cardiac injury and its possible mechanisms. We found that DOX administration significantly increased IL-16 release from cardiac macrophages. Using an antibody to neutralize IL-16 reduced the expression of several cardiac injury markers and alleviated cardiac dysfunction in DOX-induced mice. The anti-IL-16 nAb also decreased M1 macrophage-related markers and cytokine mRNA expression and decreased cardiomyocyte apoptosis in DOX-induced mice. In addition, the anti-IL-16 nAb also inhibited DOX-induced M1 macrophage differentiation and reduced cardiomyocyte apoptosis in vitro. The regulatory role of IL-16 in the inflammation involved in other systemic diseases has been widely reported, including systemic sclerosis, gouty inflammation, and MRSA pneumonia and lung injury, while its role in cardiac inflammation has been discussed less often [10, 20, 21]. In the present study, our results suggest that IL-16 can also regulate cardiac inflammation.

Common immune cells, including T lymphocytes, macrophages, and dendritic cells, are important components of the immune system. In addition to mediating an immune response, these immune cells can also release a large number of inflammatory factors, including IFN, transforming growth factor, chemokines, and ILs [22–24]. Multiple ILs, including IL-6, IL-12, and IL-22, are primarily secreted by activated immune cells and are rarely or not secreted by nonimmune cells [25–27]. Unlike these ILs, large amounts of IL-16 are secreted by both immune cells and cells [5–7]. To determine the source of IL-16, the effects of DOX administration on IL-16 expression in different cell types were examined, and the results showed that IL-16 mRNA levels were increased in CTLL-2 T lymphocytes, RAW264.7 macrophages, DC2.4 dendritic cells, and HL-1 cardiomyocytes. Our results showed that all of these cell types can secrete IL-16 and were consistent with previous studies showing that both immune and nonimmune cells are important sources of IL-16. IL-16 mRNA was elevated most significantly in RAW264.7 macrophages, and double immunofluorescence staining showed that IL-16 was produced by cardiac macrophages. These results suggest that macrophages are the most important source of IL-16 in the specific DOX-associated microenvironment.

In this study, we found that DOX administration significantly increased both cardiac and serum IL-16 expressions. In addition, IL-16 neutralization significantly alleviated the loss of BW and HW, reduced the degree of cardiomyocyte vacuolization, and decreased the expression of multiple serum cardiac injury markers in DOX-induced mice. In addition, the ultrasound and hemodynamic results showed that cardiac dysfunction in DOX-induced mice was significantly alleviated by pretreatment with the anti-IL-16 nAb. These results suggest that IL-16 neutralization can significantly reduce DOX-induced cardiac injury.

The NF-*κ*B p65 signaling pathway is one of the most common inflammation-related signaling pathways. Recently, Ye et al. reported that increased activation of the p65 pathway significantly increased M1 macrophage differentiation in both Ang II-infused and DOX-induced mice [26, 27]. Nuclear p-p65 but not total p-p65 regulates the differentiation and inflammatory response of macrophages [27–30]. To determine the effects of IL-16 on p65 pathway activation, the p-p65 levels in both the cytoplasm and nucleus were measured. The results showed that IL-16 neutralization significantly increased cytoplasmic p-p65 expression and decreased nuclear p-p65 expression in DOX-induced mice. These results suggest that the anti-IL-16 nAb reduces the DOX-induced translocation of p-p65. Ge et al. reported that p-p65 expression was increased in both the nucleus and cytoplasm in chronic animal studies [31, 32]. These conclusions may not be consistent with our results; one possible explanation is that cytoplasmic p65 is also activated by chronic inflammation. In fact, increased nuclear p-p65 expression and decreased cytoplasm p-p65 were also reported in another acute inflammatory animal model [33].

The activation of M1 macrophages and the differentiation of proinflammatory cells is mainly regulated by the p-65 signaling pathway in the nucleus, in which the nuclear expression of p-p65 promotes the differentiation of M1 macrophages [29, 30]. To further explore the mechanism by which IL-16 regulates DOX-induced cardiac injury, the differentiation of M1 macrophages was examined. The results showed that the anti-IL-16 nAb reduced the cardiac M1 macrophage marker expression, including CD80 and iNOS. The results showed that both CD80 and iNOS were reduced by the anti-IL-16 nAb in DOX-induced mice. IL-16 neutralization also inhibited the expression of a variety of DOX-induced M1 macrophage-related inflammatory factors. These results showed that IL-16 neutralization significantly reduced DOX-induced M1 macrophage differentiation and macrophage-associated proinflammatory cytokine secretion. These results also suggest that the anti-IL-16 nAb protects against DOX-induced cardiac injury by alleviating the macrophage-related inflammatory response.

Cardiomyocytes are a type of terminally differentiated cell with poor plasticity that exhibit poor tolerance to various external pathological factors, especially the inflammatory response [4]. Numerous studies have demonstrated that promoting the differentiation of M1 macrophages and amplifying the inflammatory response significantly increases DOX-induced cardiomyocyte apoptosis, while inhibiting the differentiation of M1 macrophages or promoting M2 macrophage differentiation and reducing the inflammatory response significantly reduces cardiomyocyte apoptosis [4, 34, 35]. These studies suggest that excessive cardiomyocyte apoptosis is the most fundamental mechanism of DOX-induced cardiac injury and is associated with the deterioration of cardiac function. To further explore the mechanisms involved, the effects of the anti-IL-16 nAb on DOX-induced cardiomyocyte apoptosis were examined. The results showed that IL-16 neutralization significantly reduced both the Bax/Bcl-2 ratios and cleaved caspase-3/caspase-3 ratios. In addition, fewer TUNEL-positive cells were observed in the nAb DOX group than in the IgG DOX group. These results showed that IL-16 neutralization significantly reduced cardiomyocyte apoptosis in DOX-induced mice, which suggests that IL-16 may protect against DOX-induced cardiac injury by reducing cardiomyocyte apoptosis. To verify the above conclusion, we determined the effect of the anti-IL-16 nAb on macrophage differentiation in vitro, and the results showed that IL-16 neutralization significantly inhibited the differentiation of DOX-induced M1 macrophages. HL-1 cardiomyocytes were cocultured with RAW264.7 macrophages, and the results showed that cardiomyocyte apoptosis was also mediated by macrophage differentiation. This result supports our conclusions.

In summary, our results demonstrate that IL-16 neutralization can inhibit DOX-induced M1 macrophage differentiation, reduce the inflammatory response, and decrease cardiomyocyte apoptosis, thereby alleviating cardiac injury and improving cardiac function. IL-16 neutralization may be beneficial in preventing cardiotoxicity induced by the chemotherapy drug DOX.

## Figures and Tables

**Table 1:** Real-time PCR primer sequences.

Genes	Forward primer	Reversed primer
Il-16	GCAAGACCAACTCGGTCACT	GCCCTTCATCAGCACTATGTT
TNF-*α*	CCCAGGGACCTCTCTCTAATC	ATGGGCTACAGGCTTGTCACT
IL-1*β*	GGGCCTCAAAGGAAAGAATC	TACCAGTTGGGGAACTCTGC
IL-6	AGTTGCCTTCTTGGGACTGA	TCCACGATTTCCCAGAGAAC
IL-12	AGTTTGGCCAGGGTCATTCC	TCTCTGGCCGTCTTCACCAT
IL-17	TCCAGAAGGCCCTCAGACTA	AGCATCTTCTCGACCCTGAA
IL-18	ATGCTTTCTGGACTCCTGCC	GTCTGGTCTGGGGTTCACTG
IFN-*γ*	ACTGGCAAAAGGATGGTGAC	TGAGCTCATTGAATGCTTGG
MCP-1	CTTCTGTGCCTGCTGCTCAT	CGGAGTTTGGGTTTGCTTGTC
CD80	GGCCTGAAGAAGCATTAGCTG	GAGGCTTCACCTAGAGAACCG
iNOS	TGACGCTCGGAACTGTAGCA	CAGTGATGGCCGACCTGAT
GAPDH	AACTTTGGCATTGTGGAAGG	CACATTGGGGGTAGGAACAC

**Table 2:** Data of evaluation of LV function by echocardiography and hemodynamic data of the four groups.

Group	IgG saline	nAb saline	IgG DOX	nAb DOX	Statistical method
HR (bpm)	525 ± 31	541 ± 49	449 ± 37^∗^	507 ± 34^**#**^	ANOVA
LVEF (%)	75.2 ± 2.9	76.2 ± 3.3	56.1 ± 3.4^∗^	65.9 ± 3.7^**#**^	Nonparametric test
FS (%)	40.8 ± 3.0	41.2 ± 3.2	30.8 ± 3.1^∗^	35.6 ± 3.5^**#**^	ANOVA
+dp/dt (mmHg/s)	6835 ± 909	6711 ± 716	4425 ± 684^∗^	5719 ± 845^**#**^	Nonparametric test
-dp/dt (mmHg/s)	6075 ± 734	5963 ± 598	3846 ± 496^∗^	4811 ± 695^**#**^	ANOVA

*N* = 10 for each group. ^∗^*p* < 0.05 vs. the IgG saline group. ^#^*p* < 0.05 vs. the IgG saline group.

## Data Availability

We declare that the materials described in the manuscript, including all the relevant raw data, will be freely available to any scientist wishing to use them for noncommercial purposes, without breaching participant confidentiality.
