# Corrigendum to “Anti-Interleukin-16-Neutralizing Antibody Attenuates Cardiac Inflammation and Protects against Cardiac Injury in Doxorubicin-Treated Mice”

**DOI:** 10.1155/2022/9895309

**Published:** 2022-04-29

**Authors:** Jianwei Zhang, Zicong Yang, Zhishan Liang, Mengjie Wang, Changxing Hu, Chao Chang, Lei Shi, Qingwei Ji, Ling Liu

**Affiliations:** ^1^Department of Cardiology, Beijing Anzhen Hospital, Capital Medical University, Beijing Institute of Heart, Lung, And Blood Vessel Diseases, The Key Laboratory of Remodeling-related Cardiovascular Disease, Ministry of Education, Beijing 100029, China; ^2^Department of Cardiology, The People's Hospital of Guangxi Zhuang Autonomous Region, Nanning, China; ^3^Department of Cardiology, Handan First Hospital, Handan, Hebei, China

In the article titled “Anti-Interleukin-16-Neutralizing Antibody Attenuates Cardiac Inflammation and Protects against Cardiac Injury in Doxorubicin-Treated Mice” [1], the image for IL-16 in Figure 1 has been duplicated and presented as the image for GAPDH in Figure 1.

The authors have explained that this duplication was introduced in error while uploading files during the production of their article and have highlighted the correct images have been present in their files for the duration of peer review.

The correct Figure 1 appears below.

## Figures and Tables

**Figure 1:**
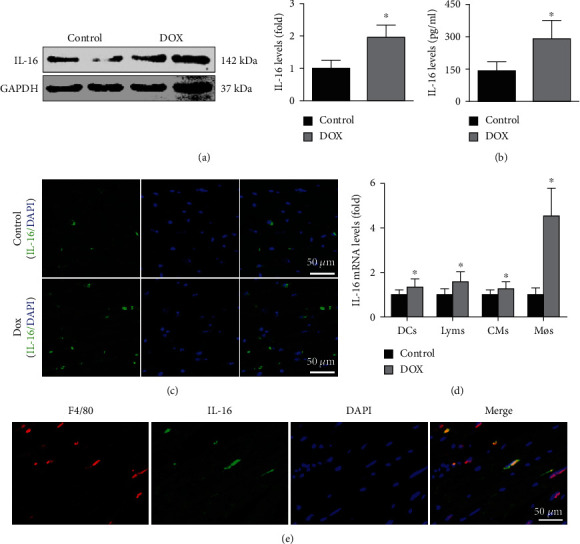
Effects of DOX on cardiac IL-16 expression. (a, b) Cardiac IL-16 expression and serum IL-16 levels were measured in the control and DOX groups (nonparametric test). (c) Cardiac IL-16 expression in the 2 groups was determined by immunofluorescence staining (200x). (d) Effects of DOX on IL-16 mRNA expression in CTLL-2 T lymphocytes (Lyms), RAW264.7 macrophages (Møs), DC2.4 dendritic cells (DCs), and HL-1 cardiomyocytes (CMs) (Student's *t*-test). (e) Double immunofluorescence staining with anti-F4/80 and anti-IL-16 in DOX-induced mice (200x). *N* = 5 in each group. ∗*p* < 0.05 vs. the control group.

## References

[1] Zhang J., Yang Z., Liang Z. (2021). Anti-Interleukin-16-Neutralizing Antibody Attenuates Cardiac Inflammation and Protects against Cardiac Injury in Doxorubicin-Treated Mice. *Mediators of Inflammation*.

